# Analyzing indirect effects in cluster randomized trials. The effect of estimation method, number of groups and group sizes on accuracy and power

**DOI:** 10.3389/fpsyg.2014.00078

**Published:** 2014-02-04

**Authors:** Joop J. Hox, Mirjam Moerbeek, Anouck Kluytmans, Rens van de Schoot

**Affiliations:** ^1^Department of Methods and Statistics, Utrecht UniversityUtrecht, Netherlands; ^2^Optentia Research Program, Faculty of HumanitiesNorth-West University, South Africa

**Keywords:** multilevel sem, sample size, cluster randomized trial, Bayesian estimation, mediation

## Abstract

Cluster randomized trials assess the effect of an intervention that is carried out at the group or cluster level. Ajzen's theory of planned behavior is often used to model the effect of the intervention as an indirect effect mediated in turn by attitude, norms and behavioral intention. Structural equation modeling (SEM) is the technique of choice to estimate indirect effects and their significance. However, this is a large sample technique, and its application in a cluster randomized trial assumes a relatively large number of clusters. In practice, the number of clusters in these studies tends to be relatively small, e.g., much less than fifty. This study uses simulation methods to find the lowest number of clusters needed when multilevel SEM is used to estimate the indirect effect. Maximum likelihood estimation is compared to Bayesian analysis, with the central quality criteria being accuracy of the point estimate and the confidence interval. We also investigate the power of the test for the indirect effect. We conclude that Bayes estimation works well with much smaller cluster level sample sizes such as 20 cases than maximum likelihood estimation; although the bias is larger the coverage is much better. When only 5–10 clusters are available per treatment condition even with Bayesian estimation problems occur.

## Introduction

With cluster randomized trials complete groups of individuals, rather than the individuals themselves, are randomized to treatment conditions. Although cluster randomized trials are less efficient than individually randomized trials, they are often preferred in practice for ethical, practical, organizational, or financial reasons, and also to lower the risk of control group contamination (Gail et al., 1996; Moerbeek, 2005). It may be considered unethical to require doctors or therapists to offer a new and promising treatment to some of their patients and to withhold it from others. In educational research it may be more cost-efficient to sample many subjects from a limited number of schools rather than to sample subjects spread over a large number of schools. Control group contamination occurs when information on the intervention leaks to the control and the risk is higher when both treatment and control conditions are available within each group.

Cluster randomized trials are common in the health and behavioral sciences, examples are school-based smoking prevention interventions, body weight reduction trials in general practices, and psychological treatments in groups. Their popularity is demonstrated by four books that are solely devoted to this design (Murray, 1998; Donner and Klar, 2000; Hayes and Moulton, 2009; Eldridge and Kerry, 2012). The main issue with cluster randomized trials is that, as subjects are nested within groups, the outcomes of subjects within the same group cannot be considered to be independent. The correct statistical model that takes such dependencies into account is generally referred to as multilevel model, mixed effects model or random coefficients model (Raudenbush and Bryk, 2002; Hox, 2010; Snijders and Bosker, 2012). This model considers groups of individuals as random effects and the results of the trial can be generalized to the population, provided the number of groups is large enough and the groups can be considered a random sample from this population. Ignoring the multilevel data structure or treating groups as fixed may result in incorrect conclusions with respect to the effect of the intervention (Moerbeek et al., 2003).

The aim of a cluster randomized trial, and of randomized controlled trials in general, is to evaluate the effect of an intervention that aims to change behavior, for instance to lower calorie intake of overweight subjects or to encourage smokers to quit smoking. The effect of the intervention on behavior may be indirect, as is stated for example by the Ajzen's theory for planned behavior (Ajzen, 1991; Conner and Armitage, 1998), which is also known as the Theory of Planned Behavior or the Theory of Reasoned Action. This model states that an individual's behavior is influenced by his or her intention, which in its turn is influenced by attitude and social norm. The latter two variables are assumed to not have a direct effect on behavior, so the effects of these variables on behavior are mediated by intention. The theory has been proven to be very effective in psychology and marketing research (Sheppard et al., 1988) but is also useful in experimental research (MacKinnon et al., 2002b). It is assumed that the intervention will affect behavior indirectly via the mediators attitude and intention. Such mediation is called chain mediation or a three path model. The variables attitude, intention and behavior are measured at the first (i.e., subject) level of the multilevel data structure while the intervention is offered at the second (i.e., group) level. The model is then a 2 → 1 → 1 → 1 model in the notation of Krull and MacKinnon (2001). Mediation can be estimated and tested using structural equation models as has been studied for models without multilevel data structures over the past decades (Sobel, 1982; Baron and Kenny, 1986; MacKinnon et al., 2002a, 2007; Shrout and Bolger, 2002; Taylor et al., 2007; MacKinnon, 2008). An extension to mediation in multilevel models has been studied more recently (Raudenbush and Sampson, 1999; Krull and MacKinnon, 2001; Bauer et al., 2006; Dagne et al., 2007; Raykov and Mels, 2007; Zhang et al., 2009; Preacher et al., 2011). A special focus on cluster randomized trials was made by Krull and MacKinnon (2001), Pituch et al. (2005, 2010), and Pituch and Stapleton (2012).

The drawback of structural equation modeling (SEM) is that it is a large sample technique while the number of clusters in a cluster randomized trial is often small, say less than fifty. This may result in estimation problems when maximum likelihood estimation is used, such as convergence problems, inadmissible estimates such as negative variances, biased chi-squared test statistics and standard errors and low statistical power. Furthermore, it is less robust against non-normality, which is a problem in mediation models where the indirect effect is not normally distributed. Bayesian estimation methods do not assume large sample sizes (Gelman et al., 2004), and in mediation analysis have the additional advantages that they directly incorporate the nonnormal mediation effect, and that they are conceptually simpler for multilevel mediation models (Yuan and MacKinnon, 2009).

Simulation studies have shown that using fewer than 50 clusters is problematic while using maximum likelihood estimation (Maas and Hox, 2005; Meuleman and Billiet, 2009; Hox et al., 2010). A recent simulation study suggested that a much lower sample size at the cluster level of approximately 20 is sufficient when Bayesian estimation methods are used (Hox et al., 2012). The model used in this study did not include mediation effects, though.

The aim of our paper is to study the lowest number of clusters that is needed to accurately estimate and test mediation in cluster randomized trials. We focus on convergence problems, accuracy of the point estimate and confidence interval and power of the test on the indirect effect from treatment condition to behavior. We compare maximum likelihood to Bayesian methods and expect the latter to perform better as it does not assume large sample sizes and normality. Furthermore, since in Bayesian estimation the parameter estimates all follow their proper distribution, we expect no inadmissible solutions.

## Statistical model and estimation methods

**Figure 1:**
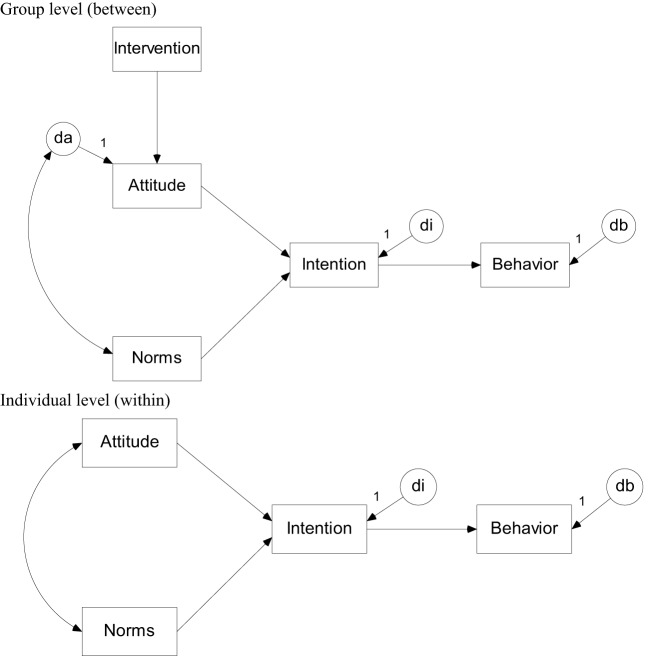
**The planned behavior model at the between-cluster level (Top panel) and within-cluster level (Bottom panel)**.

Subjects are nested within clusters and for both the within-and between level a structural equation model is formulated, see Figure 1. Treatment condition is a cluster-level variable and only appears at the between-level. It affects behavior only indirectly through attitude and intention. All other variables are measured at the subject level are assumed to vary both within and between clusters, hence they are included in both the within- and between-level model. Variables that are predicted from one or more others are called endogenous variables and require a disturbance term to capture unexplained variance. For the variable attitude a disturbance term only appears at the between-level. This variable is assumed to correlate with norms at both levels. The numbers in Figure 1 refer to the population values in our simulation, which is discussed in the next section.

Multilevel Structural Equation Modeling (MSEM) assumes sampling at both individual and group levels. The individual data are collected in a *p*-variate vector **Y**_*ij*_ with subscript *i* for individuals and *j* for groups. The data **Y**_*ij*_ are decomposed into a group level (between groups) component **Y**_*B*_ = **Y**_*j*_ and an individual level (within groups) component **Y**_*W*_ = **Y**_*ij*_ − **Y**_*j*_. These two components are orthogonal and additive: **Y**_*T*_ = **Y**_*B*_ + **Y**_*W*_, and the population covariance matrices are also orthogonal and additive: Σ_*T*_ = Σ_*B*_ + Σ_*W*_. MSEM assumes that the population covariance matrices Σ_*B*_ and Σ_*W*_ are described by distinct models for the group level and the individual level structure. Full maximum likelihood estimation for MSEM minimizes the fit function given by

(1) $$F=\sum_{i=1}^{N}\log\left|\Sigma_{i}\right|+\sum_{i=1}^{N}\log{\left(x_{i}-\mu_{i}\right)}^{\text{'}}\Sigma_{i}^{-1}\left(x_{i}-\mu_{i}\right)\text{​},$$

where the subscript *i* refers to the observed cases, **x**_*i*_ to those variables observed for case *i*, and μ_*i*_ and Σ_*i*_ contain the population means and covariances of the variables observed for case *i* (cf. Mehta and Neale, 2005).

Maximum likelihood estimation assumes large samples, and relies on numerical methods to integrate out random effects. In comparison, Bayesian methods are reliable in small samples, and deal better with complex models. The Bayesian approach is fundamentally different from classical statistics (Barnett, 2008). In classical statistics, the population parameter has one specific value that is unknown. In Bayesian statistics, we express the uncertainty about the population value of a model parameter by specifying a probability distribution of possible values. This probability distribution is called the *prior* distribution, because it is specified independently from the data. After we have collected our data, this prior distribution is combined with the Likelihood of the data to produce a *posterior* distribution, which describes our uncertainty about the population values after observing our data. Typically, the variance of the posterior distribution is smaller than the variance of the prior distribution, which means that observing the data has reduced our uncertainty about the possible population values.

Obtaining the posterior distribution is done by simulation, using so-called *Markov Chain Monte Carlo (MCMC)* methods. The general idea of MCMC is that instead of attempting to analytically solve for the point estimates, as with ML estimation, an iterative simulation procedure is used to estimate the parameters. It is beyond the scope of the current paper to provide a full introduction to Bayesian modeling, we refer the non-informed reader to, among many others, Kruschke (2011), van de Schoot et al. (2013) and for a more technical introduction see Gelman et al. (2004) or Lynch (2007). For Bayesian multilevel modeling we refer to Hamaker and Klugkist (2011) and Fahrmeir et al. (2013).

The mode of the marginal posterior distribution is an attractive point estimate of the unknown parameter, because it is the most likely value, and therefore the Bayesian equivalent of the maximum likelihood estimator. Since the mode is more difficult to determine than the mean, the mean of the posterior distribution is also often used. In skewed posterior distributions, the median is an attractive choice. In Bayesian estimation, the standard deviation of the posterior distribution is comparable to the standard error in classical statistics. However, the confidence interval generally is based on the ½α and 100-½α percentiles around the point estimate. In the Bayesian terminology, this is referred to as the 100-α% *credible interval*. We will return to the interpretation of frequentist and Bayesian intervals in the discussion. Given the non-normality of the mediation effect, we have chosen to use the median of the posterior distribution for the point estimate, and the percentile-based 95% credible interval.

Bayesian methods have some advantages over classical methods. As mentioned before, in contrast to the asymptotic maximum likelihood method, they are valid in small samples. Given the correct probability distribution, the estimates are always proper, which solves the problem of negative variance estimates. Finally, since the random draws are taken from the correct distribution, there is no assumption of normality when variances are estimated. In this study, we examine if Bayesian estimation will help in drawing correct inferences in multilevel SEM if the number of groups (clusters) is relatively small. The simulation studies cited in the introduction typically find that at smaller group level sample sizes the Maximum Likelihood based parameter estimates themselves are unbiased, but that the corresponding standard errors are underestimated, which leads to poor control of the alpha level and undercoverage for the confidence intervals. We expect that the credible intervals in our Bayesian estimation will perform better at lower group level sample sizes.

## Design of the simulation study

**Figure A1:**
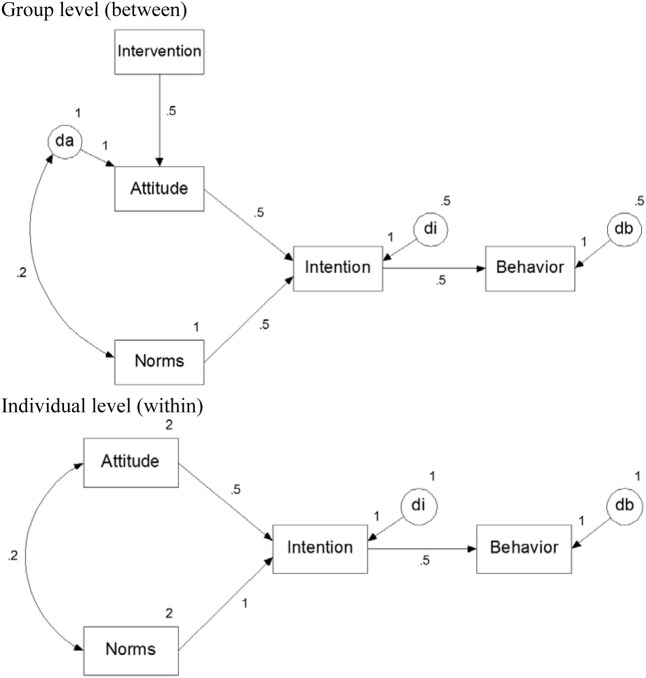
**The planned behavior model at the between-cluster level (Top panel) and within-cluster level (Bottom panel) with population parameter values**.

Data are simulated on basis of the model in Figure 1. The number of clusters per treatment condition was 5, 10, 25, or 50. The cluster size was either 5 or 10. The eight combinations of the sample sizes at the cluster and subject level are henceforth called populations and for each of them 5000 data sets were generated using the program Mplus 7.0 (Muthén and Muthén, 2012). Treatment condition was a dichotomous variable whereas attitude, norms, intention and behavior were all multivariate normally distributed. The effect of treatment on attitude was set at 0.5. All mediation paths at the within-level were set to the medium effect size 0.5, which implies that the mediation effect is 0.125. The error variance of the endogenous variable attitude was fixed to 2 at the within-level and 1 at the between-level. The error variances of intention and behavior were fixed to 1 and 0.5 at the within- and between-level. Hence, the intra-class correlation coefficients for all three endogenous variables were 0.33. The population values used in the simulation are presented in the path model in Figure A1.

The program Mplus was also used for model parameter estimation. Default convergence criteria were used for maximum likelihood estimation but not for Bayesian estimation. For the Bayesian estimation we used the Mplus 7.0 default priors which are a very flat normal distribution (N(0,10^10^)) for the path coefficients and a flat inverse gamma distribution (IG(−1,0)) for the variances. By default, Mplus 7.0 uses two independent MCMC chains, and uses the Gelman-Rubin potential scale reduction (PSR; Gelman and Rubin, 1992) to assess convergence. Convergence is reached when the PSR criterion is less than 0.05 from 1. In our simulation, we have run four independent MCMC chains, forced a chain length of at least 5000, and set the PSR criterion to 0.01 for convergence to be reached. For the point estimate we used the median of the posterior, and for the 95% credible interval we used the percentile method.

The performance of the two estimation methods was based on four criteria: convergence, parameter bias, coverage of the 95% confidence interval (maximum likelihood estimation) or 95% credible interval (Bayes estimation) and power. Our focus is on the mediation effect from treatment condition to mediation.

## Results

Table 1 displays the mean, (standard deviation), relative bias, coverage of the 95% CI, and percentage significant results over 5000 replications per cell for the mediation effect from treatment condition to mediation.

**Table 1:** **Simulation study results for the eight populations using Maximum Likelihood and Bayes**.

**Estimator**	**Outcome**	**Pop 1 50 (5) 50 (5)**	**Pop 2 50 (10) 50 (10)**	**Pop 3 25 (5) 25 (5)**	**Pop 4 25 (10) 25 (10)**	**Pop 5 10 (5) 10 (5)**	**Pop 6 10 (10) 10 (10)**	**Pop 7 5 (5) 5 (5)**	**Pop 8 5 (10) 5 (10)**
ML	Mean (*SD*)	0.1247	0.1256	0.1249	0.1244	0.1249	0.1259	0.1238	0.1242
		(0.0713)	(0.0624)	(0.1105)	(0.0902)	(0.7643)	(0.1730)	(0.7178)	(0.4009)
	Bias	−0.24	0.48	−0.08	−0.48	−0.08	0.72	−0.96	−0.64
	95% Coverage	92%	92.9%	90.4%	91%	90.8%	88.6%	92%	89.6%
	5% Significance	33.1%	51.8%	9.3%	16.8%	2.8%	4.8%	4.2%	4.8%
Bayes	Mean (*SD*)	0.1125	0.1165	0.1046	0.1092	0.0869	0.0965	0.0662	0.0716
		(0.0753)	(0.0688)	(0.1152)	(0.0992)	(0.2392)	(0.2067)	(0.6044)	(0.5281)
	Bias	−10	−6.8	−16.32	−12.64	−30.48	−22.8	−47.04	−42.72
	95% Coverage	94.6%	95.2%	95.4%	94.5%	99.2%	97.9%	100%	99.9%
	5% Significance	50.3%	59.7%	15.2%	25.4%	1.2%	3.8%	0.1%	0.1%

Contrary to expectations, the Bayesian estimation ran into biased estimates for all eight populations' mediation effect whereas the Maximum Likelihood estimation was able to provide only slightly biased estimates for the mediation effect so long as there were about 50 clusters with 5–10 observations each. However, as a consequence of the biased standard errors in ML estimation, the 95% coverage and significance intervals for the Bayesian estimation outperformed those of the Maximum Likelihood estimation. Bayesian estimation rendered overall higher coverage rates and higher significance rates for the first four populations—until the number of clusters drops below 25.

Table 2 displays information about the convergence of the analyses for the Maximum Likelihood estimation. The pattern in Table 2 suggests that as soon as the number of clusters drops below 25, Maximum Likelihood runs into considerable convergence problems. The types of convergence warnings are displayed below Table 2; their frequencies (if any) are displayed in the final column of the table.

**Table 2:** **Convergence in the simulation study with ML estimation**.

**Pop**	**Requested**	***N* Incomplete**	**% Incomplete**	**Complete**	**Warnings**	**% Warnings**	Warning types^*^
1, 50:5	5000	0	0	5000	0	0	–
2, 50:10	5000	0	0	5000	0	0	–
3, 25:5	5000	0	0	5000	2	0.04	2^*^1
4, 25:10	5000	0	0	5000	0	0	–
5, 10:5	5000	3	0.06	4997	2077	41.565	165^*^1, 1912^*^2, 1^*^4 + 3
6, 10:10	5000	0	0	5000	1734	34.68	14^*^1, 1720^*^2
7, 5:5	5000	1596	31.92	3404	3404	100	3404^*^2
8, 5:10	5000	543	10.86	4457	4457	100	4457^*^2

* *Warning types*.

*1. Warning: The MLR standard errors could not be computed. The MLF standard errors were computed instead. The MLR condition number is −0.463D-03. Problem involving parameter 17. This may be due to near of the random effect variance/covariance or incomplete convergence singularity*.

*2. The standard errors of the model parameter estimates may not be trustworthy for some parameters due to a non-positive definite first-order derivative product matrix. This may be due to the starting values but may also be an indication of model non-identification. The condition number is 0.820D-11. Problem involving parameter 20. The non-identification is most likely due to having more parameters than the number of clusters. reduce the number of parameters*.

*3. The model estimation did not terminate normally due to an ill-conditioned fisher information matrix. Change your model and/or starting values. The model estimation did not terminate normally due to a non-positive definite fisher information matrix. This may be due to the starting values but may also be an indication of model non-identification. The condition number is 0.371D-15. The standard errors of the model parameter estimates could not be computed. This is often due to the starting values but may also be an indication of model non-identification. Change your model and/or starting values. Problem involving parameter 20*.

*4. One or more parameters were fixed to avoid singularity of the information matrix. The singularity is most likely distribution of the categorical variables in the model. Model is not identified, or because of empty cells in the joint because the following parameters were fixed: 21*.

## Discussion

The simulation results indicate that Bayesian estimation works better at smaller second level sample sizes than Maximum Likelihood estimation. Actually, Bayesian estimation results in a larger bias than Maximum Likelihood estimation at all simulated sample sizes. However, the standard errors in the ML estimation were inaccurate at the lower sample sizes, and as a result the Bayesian 95% CI shows a much better coverage than the ML 95% CI. This is not only the result of having more accurate standard errors, but also due to the fact that the mediation effect is a multiplication of effects that are assumed to be normal, and therefore the mediation effect does not follow a normal distribution. The Bayesian posterior 95% credible interval is established by the percentile method, which follows the asymmetric distribution of the mediation effect much better than the symmetric intervals established by the Maximum Likelihood method (Yuan and MacKinnon, 2009). The ML estimation method shows a better power, but at the expense of having standard errors that are estimated with a downward bias, resulting in poor type I error control due to an operating alpha level which is higher than the nominal 5% level.

At the smaller sample sizes ML estimation exhibits convergence problems, as expected. Bayesian estimation can also encounter estimation problems, but of a different kind. In MCMC estimation, convergence means convergence of the chain to the correct distribution. In our simulation we must rely on the automatic convergence criteria available in Mplus. Textbooks on Bayesian statistics caution users to always use diagnostic tools such as plots of the iteration history (trace plots, cf. Gelman et al., 2004; Lynch, 2007), and we completely agree with these recommendations; we consider such inspections mandatory. Especially with small sample sizes, we recommend inspection of autocorrelations and setting much stricter criteria for convergence. In addition, with smaller sample sizes, the use of informative priors could be helpful. The disadvantage is of course, that in small samples such prior information can easily dominate the information in the data. Here, we have taken the position that this is undesirable, and prefer to work with uninformative priors. If informative priors are to be used, we recommend using several of these priors to determine to what extend the choice of a specific prior determines the results.

In the section on the model and estimation method we introduced the frequentist 95% confidence interval and the Bayesian 95% credible interval, tacitly implying that these are more or less the same (they both abbreviate to 95% CI). The software used (Mplus 7; Muthén and Muthén, 2012) encourages this view since to employ Bayesian estimation we simply have to specify a different estimation method. We do think that users should realize that by choosing Bayesian estimation, from a principled standpoint, they have chosen to employ a different kind of statistics. As a result, the 95% credibility interval now may correctly be interpreted as the interval that contains the population parameter with 95% probability. In our power section in Table 1, we refer to significance of results, based on frequentist and Bayesian. *p*-values. In the Bayesian case, this is not the identical to the frequentist *p*-value, it is the so-called posterior predictive *p*-value. This is roughly interpreted as a standard *p*-value, but it is actually a different entity. Bayesian modelers in general prefer that decisions about parameters are based on credibility intervals and not *p*-values, and that decisions about models are based on comparative evidence, such as information criteria or Bayes factors. A discussion of these issues is beyond the scope of this paper (for a very thorough discussion see Barnett, 2008), but we believe that applied researchers should be aware that doing a Bayesian analysis is not just choosing a different estimation method.

One of the reviewers raised the issue that, since the mediation effect is a product term of three coefficients, the priors that are specified to be uninformative on the coefficients themselves, may become informative on the indirect mediation effect. This concern is quite right. The default prior for regression coefficients in Mplus 7 is a normal distribution with a mean of zero and a very large variance. The distribution of products of random variables is generally hard to assess (see Glen et al., 2004, for a discussion and a computational approach). When the normal distributions have a mean of zero, the product of three normally distributed variables is a complicated function that is symmetric with a peak at zero (see the explication on the Mathematica website at http://mathworld.wolfram.com/NormalProductDistribution.html). It appears that, even if the standard deviation of the prior normal distributions are very large, there is still a real possibility that their convolution is informative, and will tend to move the posterior toward zero. The fact that the Bayesian estimates reported in Table 1 have a downward bias in all simulated conditions suggests that this is in our simulation setup indeed the case. Using uniform priors, although their convolution also does not lead to a uniform prior for the mediation effect, may ameliorate the situation by producing a less peaked prior for the mediation effect. This is clearly an area that needs further research.

### Conflict of interest statement

The authors declare that the research was conducted in the absence of any commercial or financial relationships that could be construed as a potential conflict of interest.

## References

[B1] Ajzen I. (1991). The theory of planned behavior. Organ. Hum. Dec. Process. 50, 179–211 10.1016/0749-5978(91)90020-T

[B2] Barnett V. (2008). Comparative Statistical Inference. Chicester: Wiley

[B3] Baron R. M. Kenny D. A. (1986). The moderator–mediator variable distinction in social psychological research: conceptual, strategic, and statistical considerations. J. Pers. Soc. Psychol. 51, 1173–1182 10.1037/0022-3514.51.6.1173 3806354

[B4] Bauer D. J. Preacher K. J. Gil K. M. (2006). Conceptualizing and testing random indirect effects and moderated mediation in multilevel models: new procedures and recommendations. Psychol. Methods 11, 142–163 10.1037/1082-989X.11.2.142 16784335

[B5] Conner M. Armitage C. J. (1998). Extending the theory of planned behavior: a review and avenues for further research. J. Appl. Soc. Psychol. 28, 1429–464 10.1111/j.1559-1816.1998.tb01685.x

[B6] Dagne G. A. Hendricks Brown C. Howe G. W. (2007). Hierarchical modeling of sequential behavioral data: examining complex association patterns in mediation models. Psychol. Methods 12, 298–316 10.1037/1082-989X.12.3.298 17784796

[B7] Donner A. Klar N. (2000). Design and Analysis of Cluster Randomization Trials in Health Research. London: Edward Arnold

[B8] Eldridge M. Kerry S. (2012). A Practical Guide to Cluster Randomized Trials in Health Services Research. Chichester: Wiley 10.1002/9781119966241

[B9] Fahrmeir L. Kneib T. Lang S. (2013). Bayesian multilevel models, in The Sage Handbook of Multilevel Modeling, eds Scott M. A. Simonoff J. S. Marx B. D. (Los Angeles, CA: Sage), 53–72 10.4135/9781446247600.n4

[B10] Gail M. Mark S. Carroll R. Green S. (1996). On design considerations and randomization-based inference for community intervention trials. Stat. Med. 15, 1069–1092 10.1002/(SICI)1097-0258(19960615)15:11<1069::AID-SIM220>3.0.CO;2-Q 8804140

[B11] Gelman A. Carlin J. B. Stern H. S. Rubin D. B. (2004). Bayesian Data Analysis, 2nd Edn. Boca Raton, FL: Chapman and Hall/CRC

[B12] Gelman A. Rubin D. B. (1992). Inference from iterative simulation using multiple sequences. Stat. Sci. 7, 457–511 10.1214/ss/1177011136

[B13] Glen A. G. Leemis L. M. Drew J. H. (2004). Computing the distribution of the product of two continuous random variables. Comput. Stat. Data Anal. 44, 451–464 10.1016/S0167-9473(02)00234-7

[B14] Hamaker E. L. Klugkist I. (2011). Bayesian estimation of multilevel models, in Handbook of Advanced Multilevel Analysis, eds Hox J. J. Roberts J. K. (New York, NY: Routledge), 137–162

[B15] Hayes R. J. Moulton L. H. (2009). Cluster Randomised Trials. Boca Rotan, FL: CRC Press 10.1201/9781584888178

[B16] Hox J. J. (2010). Multilevel Analysis. Techniques and Applications, 2nd Edn. New York, NY: Routledge

[B17] Hox J. J. Maas C. J. M. Brinkhuis M. J. S. (2010). The effect of estimation method and sample size in multilevel structural equation modelling. Stat. Neerl. 64, 157–170 10.1111/j.1467-9574.2009.00445.x

[B18] Hox J. van de Schoot R. Matthijsse S. (2012). How few countries will do? Comparative survey analysis from a Bayesian perspective. Surv. Res. Methods 6, 87–93

[B19] Kruschke J. K. (2011). Doing Bayesian Data Analysis. Burlingon, MA: Academic Press

[B20] Krull J. L. MacKinnon D. P. (2001). Multilevel modelling of individual and group level mediated effects. Multivariate Behav. Res. 36, 249–277 10.1207/S15327906MBR3602_06 26822111

[B21] Lynch S. (2007). Introduction to Applied Bayesian Statistics and Estimation for Social Scientists. New York, NY: Springer 10.1007/978-0-387-71265-9

[B22] Maas C. J. M. Hox J. J. (2005). Sufficient sample sizes for multilevel modelling. Methodology 1, 86–92 10.1027/1614-2241.1.3.85

[B23] MacKinnon D. P. (2008). Statistical Mediation Analysis. New York, NY: Lawrence Erlbaum Associates

[B24] MacKinnon D. P. Fairchild A. J. Fritz M. S. (2007). Mediation analysis. Annu. Rev. Psychol. 58, 593–614 10.1146/annurev.psych.58.110405.085542 16968208 PMC2819368

[B25] MacKinnon D. P. Lockwood C. M. Hoffman J. M. West S. G. Sheets V. (2002a). A comparison to test mediation and other intervening variable effects. Psychol. Methods 7, 83–104 10.1037/1082-989X.7.1.83 11928892 PMC2819363

[B26] MacKinnon D. P. Taborga M. P. Morgan–Lopez A. A. (2002b). Mediation designs for tobacco prevention research. Drug Alcohol Depend. 68, 569–583 10.1016/S0376-8716(02)00216-8 12324176 PMC2821204

[B27] Mehta P. D. Neale M. C. (2005), People are variables too: multilevel structural equations modeling. Psychol. Methods 10, 259–284 10.1037/1082-989X.10.3.259 16221028

[B28] Meuleman B. Billiet J. (2009). A Monte Carlo sample size study: how many countries are needed for accurate multilevel SEM? Surv. Res. Methods 3, 45–58

[B29] Moerbeek M. (2005). Randomization of clusters versus randomization of persons within clusters: which is preferable? Am. Stat. 59, 72–78 10.1198/000313005X20727

[B30] Moerbeek M. Van Breukelen G. J. P. Berger M. P. F. (2003). A comparison between traditional methods and multilevel regression for the analysis of multi-center intervention studies. J. Clin. Epidemiol. 56, 341–350 10.1016/S0895-4356(03)00007-6 12767411

[B31] Murray D. M. (1998). Design and Analysis of Group-Randomized Trials. New York, NY: Oxford University Press

[B32] Muthén L. K. Muthén B. O. (2012). Mplus User's Guide, 7th Edn. Los Angeles, CA: Muthén and Muthén

[B33] Pituch K. A. Murphy D. L. Tate R. L. (2010). Three-level models for indirect effects in school- and class-randomized experiments in education. J. Exp. Educ. 78, 60–95 10.1080/00220970903224685

[B34] Pituch K. A. Stapleton L. M. (2012). Distinguishing between cross- and cluster-level mediation processes in the cluster randomized trial. Sociol. Methods Res. 41, 630–670 10.1177/0049124112460380

[B35] Pituch K. A. Whittaker T. A. Stapleton L. A. (2005). A comparison of methods to test for mediation in multisite experiments. Multivariate Behav. Res. 40, 1–23 10.1207/s15327906mbr4001_1 26822271

[B36] Preacher K. J. Zhang Z. Zyphur M. J. (2011). Alternative methods for assessing mediation in multilevel data: the advantages of multilevel SEM. Struct. Equ. Model. 18, 161–182 10.1080/10705511.2011.557329

[B37] Raudenbush S. W. Bryk A. (2002). Hierarchical Linear Models. Applications and Data Analysis Methods. Thousand Oaks, CA: Sage Publications

[B38] Raudenbush S. W. Sampson R. (1999). Assessing direct and indirect effects in multilevel designs with latent variables. Sociol. Methods Res. 28, 123–153 10.1177/0049124199028002001

[B39] Raykov T. Mels G. (2007). Lower level mediation effect analysis in two- level multilevel structural equation modeling approach. Struct. Equ. Model. 14, 636–648 10.1080/10705510701575511

[B40] van de Schoot R. Kaplan D. Denissen J. Asendorpf J. B. Neyer F. J. van Aken M. A. G. (2013). A gentle introduction to bayesian analysis: applications to research in child development. Child Dev. [Epub ahead of print]. 10.1111/cdev.12169 24116396 PMC4158865

[B41] Sheppard B. H. Hartwick J. Warshaw P. R. (1988). The theory of reasoned action: a meta- analysis of past research with recommendations for modifications and future research. J. Consum. Res. 15, 325–343 10.1086/209170

[B42] Shrout P. E. Bolger N. (2002). Mediation in experimental and nonexperimental studies: new procedures and recommendations. Psychol. Methods 7, 422–445 10.1037/1082-989X.7.4.422 12530702

[B43] Snijders T. A. B. Bosker R. J. (2012). Multilevel Analysis: An Introduction to Basic and Advanced Multilevel Modeling. London: Sage

[B44] Sobel M. E. (1982). Asymptotic confidence intervals for indirect effects in structural Equation models. Sociol. Methodol. 13, 290–312 10.2307/270723

[B45] Taylor A. B. MacKinnon D. P. Tein J.-Y. (2007). Test of the three-path mediated effect. Organ. Res. Methods 11, 241–269 10.1177/1094428107300344 23077662

[B46] Yuan Y. MacKinnon D. P. (2009). Bayesian mediation analysis. Psychol. Methods 14, 301–322 10.1037/a0016972 19968395 PMC2885293

[B47] Zhang Z. Zyphur M. J. Preacher K. J. (2009). Testing multilevel mediation using hierarchical linear models. Problems and solutions. Organ. Res. Methods 12, 695–719 10.1177/1094428108327450

